# The use of erythropoiesis-stimulating agents with ruxolitinib in patients with myelofibrosis in COMFORT-II: an open-label, phase 3 study assessing efficacy and safety of ruxolitinib versus best available therapy in the treatment of myelofibrosis

**DOI:** 10.1186/s40164-015-0021-2

**Published:** 2015-09-15

**Authors:** Mary Frances McMullin, Claire N. Harrison, Dietger Niederwieser, Hilde Demuynck, Nadja Jäkel, Prashanth Gopalakrishna, Mari McQuitty, Viktoriya Stalbovskaya, Christian Recher, Koen Theunissen, Heinz Gisslinger, Jean-Jacques Kiladjian, Haifa-Kathrin Al-Ali

**Affiliations:** Center for Cancer Research and Cell Biology, Queen’s University, Belfast, UK; Guy’s and St. Thomas’ NHS Foundation Trust, London, UK; University of Leipzig, Leipzig, Germany; H. Hartziekenhuis, Roeselare, Belgium; Novartis Pharma AG, Basel, Switzerland; Institut Universitaire du Cancer de Toulouse, Université de Toulouse III, Toulouse, France; Limburgs Oncologisch Centrum, Hasselt, Belgium; Medical University of Vienna, Vienna, Austria; Hôpital Saint-Louis et Université Paris Diderot, Paris, France

**Keywords:** Myelofibrosis, Ruxolitinib, Anemia management, Erythropoiesis-stimulating agents, ESA

## Abstract

**Background:**

Anemia is considered a negative prognostic risk factor for survival in patients with myelofibrosis. Most patients with myelofibrosis are anemic, and 35–54 % present with anemia at diagnosis. Ruxolitinib, a potent inhibitor of Janus kinase (JAK) 1 and JAK2, was associated with an overall survival benefit and improvements in splenomegaly and patient-reported outcomes in patients with myelofibrosis in the two phase 3 COMFORT studies. Consistent with the ruxolitinib mechanism of action, anemia was a frequently reported adverse event. In clinical practice, anemia is sometimes managed with erythropoiesis-stimulating agents (ESAs). This post hoc analysis evaluated the safety and efficacy of concomitant ruxolitinib and ESA administration in patients enrolled in COMFORT-II, an open-label, phase 3 study comparing the efficacy and safety of ruxolitinib with best available therapy for treatment of myelofibrosis. Patients were randomized (2:1) to receive ruxolitinib 15 or 20 mg twice daily or best available therapy. Spleen volume was assessed by magnetic resonance imaging or computed tomography scan.

**Results:**

Thirteen of 146 ruxolitinib-treated patients had concomitant ESA administration (+ESA). The median exposure to ruxolitinib was 114 weeks in the +ESA group and 111 weeks in the overall ruxolitinib arm; the median ruxolitinib dose intensity was 33 mg/day for each group. Six weeks before the first ESA administration, 10 of the 13 patients had grade 3/4 hemoglobin abnormalities. These had improved to grade 2 in 7 of the 13 patients by 6 weeks after the first ESA administration. The rate of packed red blood cell transfusions per month within 12 weeks before and after first ESA administration remained the same in 1 patient, decreased in 2 patients, and increased in 3 patients; 7 patients remained transfusion independent. Reductions in splenomegaly were observed in 69 % of evaluable patients (9/13) following first ESA administration.

**Conclusions:**

Concomitant use of an ESA with ruxolitinib was well tolerated and did not affect the efficacy of ruxolitinib. Further investigations evaluating the effects of ESAs to alleviate anemia in ruxolitinib-treated patients are warranted (ClinicalTrials.gov identifier, NCT00934544; July 6, 2009).

## Background

Myelofibrosis (MF) is a clonal neoplastic disease characterized by bone marrow fibrosis, splenomegaly, and debilitating constitutional symptoms (fever, weight loss, night sweats) [1–4]. Dysregulation of the Janus kinase (JAK) pathway is considered to be the primary mechanism responsible for the pathophysiology of MF [5], which results in clonal myeloproliferation associated with bone marrow fibrosis, extramedullary hematopoiesis, and abnormal cytokine expression [6]. Progressive bone marrow fibrosis leads to several cytopenias, particularly thrombocytopenia and anemia [7]. MF-associated anemia is multifactorial [8] and is considered a negative prognostic risk factor for survival [1]. Between 35 and 54 % of patients present with anemia at diagnosis, with the proportion increasing to 47–64 % after 1 year or more from MF diagnosis [8–10]. Ruxolitinib is a potent JAK1 and JAK2 inhibitor that has demonstrated rapid and durable reductions in splenomegaly and improved symptoms and quality of life measures in the two phase 3 Controlled Myelofibrosis Study with Oral JAK Inhibitor Treatment (COMFORT) trials [11, 12]. Ruxolitinib-treated patients experienced prolonged survival compared with placebo (COMFORT-I) [11, 13] and best available therapy (BAT; COMFORT-II) [12], suggesting an overall survival benefit associated with ruxolitinib treatment. Consistent with the mechanism of action of ruxolitinib and the pathophysiology of MF, anemia (hemoglobin level <100 g/L) was one of the most frequently reported adverse events (AEs) but was generally manageable; only 2 of 146 patients (1 %) in COMFORT-II discontinued due to anemia after approximately 3 years of treatment (median follow-up, 151 weeks) [14].

Findings from both COMFORT studies suggest that low hemoglobin levels may be an initial concern with ruxolitinib but that hemoglobin levels increase with longer-term treatment. In COMFORT-II, ruxolitinib-treated patients experienced an initial decrease in mean hemoglobin levels over the first 12 weeks, but levels recovered to levels similar to those in the BAT arm and remained greater than 100 g/L from week 24 onward (>151 weeks) [14]. These data mirrored those from COMFORT-I in which mean hemoglobin levels in the ruxolitinib arm reached a nadir at approximately 8–12 weeks and recovered to a new steady state by week 24, independent of transfusions or dose reduction [3, 11]. Additionally, long-term follow-up of COMFORT-I showed that the incidence of new-onset grade 3/4 anemia decreased with longer-term therapy [13]. These data, in concert with the observed reduction in transfusion rate in transfusion-dependent patients, suggest that anemia in patients taking ruxolitinib is generally not a long-term safety concern.

In COMFORT-II, anemia was primarily managed with packed red blood cell (PRBC) transfusions. In clinical practice, anemia can also be managed with erythropoiesis-stimulating agents (ESAs; e.g., darbepoetin alfa, epoetin alfa) [15], which improve hemoglobin levels and may eliminate the need for transfusions [15]. In patients with MF, ESAs can be ineffective, especially if patients are transfusion dependent or have elevated erythropoietin levels [16–18]. In COMFORT-II, ESA use was discouraged (although not prohibited) because ESAs can activate the JAK pathway, potentially resulting in increased spleen size and thereby confounding efficacy analyses of spleen response [15, 19]. A small number of patients (13/146), however, received both an ESA and ruxolitinib. This report evaluates the safety and efficacy of concomitant ruxolitinib and ESA administration in these 13 patients.

## Results and discussion

To aid in the management of anemia, ESAs were administered to 13 of 146 ruxolitinib-treated patients (9 %; +ESA group; darbepoetin alfa, n = 3; epoetin alfa, n = 9; unspecified epoetin, n = 1). ESA schedules varied and doses ranged from 10,000 to 40,000 units for epoetin alfa; 40–300 μg, 150–300 μg, and 500 μg in the 3 patients receiving darbepoetin alfa; and 10,000–20,000 IU for unspecified epoetin. The median exposure to ruxolitinib was similar between the +ESA group (114 weeks) and the overall ruxolitinib arm (111 weeks), and the median ruxolitinib dose intensity was the same for both groups (33 mg/day). In the +ESA group, 11 patients (84.6 %) had dose reductions or interruptions of ruxolitinib—a rate that was similar to that in the overall study population at the time of the primary analysis (71.2 %). At study baseline, and prior to treatment with ruxolitinib or an ESA, patients who received concomitant ESAs presented with low hemoglobin levels [median hemoglobin level, 92 g/L (range 83–144) vs 106.0 g/L (range 65–162) in the ruxolitinib arm; median reticulocyte count, 138 × 10^9^/L (range 96.0–141.0) vs 121.85 × 10^9^/L (range 9.3–283.2), respectively; Table 1] and showed no signs of renal impairment [median creatinine level, 70.2 µmol/L (range 55.0–134.7) vs 75.25 µmol/L (range 38.8–172.5)], indicating anemia was not due to a combination of chronic kidney disease and erythropoietin deficiency. Comparative statistics between the overall ruxolitinib arm and +ESA group were not possible due to the small sample size of the +ESA group.

**Table 1:** Transfusion rates, hemoglobin levels, reticulocyte counts, and changes in spleen volume before and after ESA

Pt	Transfusion rate, units PRBC/month			Hemoglobin value, g/L			Reticulocyte count, ×10^9^/L			Spleen volume, L		
	BL	Before ESA^a^	After ESA^b^	BL	Before ESA^c^	After ESA^d^	BL	Before ESA^c^	After ESA^d^	BL	Change from BL (%)	
											Before ESA^e^	After ESA^f^
1	0	2.9	2.9	106	62	69	116.9	42.5	41	2.48	−13	−20
2	0.7	2.2	10.7	99	75	60	144.9	110	114.3	3.47	−22	NA
3	0.7	1.8	0.7	100	62	NA	162.1	90	NA	2.29	−29	NA
4	0	0	0.7	121	68	66	98.1	61	64	1.79	−8	−15
5	2.9	2.9	0	90	74	40	96	167	120.3	3.43	+11	−3
6	1.4	0.7	2.2	83	67	73	81.1	74	NA	3.07	−3	NA
7	0	0	0	144	78	88	156.4	39	96.1	3.04	−36	−50
8	0	0	0	93	81	80	138	82	73.4	5.24	−45	−34
9	0	0	0	90	76	78	66.9	42	42.5	2.53	−65	−67
10	0	0	0	86	74	91	92.1	49	75.7	0.46	−54	−58
11	0	0	0	92	76	73	141	106	74.9	3.16	−13	−20
12	0	0	0	90	87	84	283.2	247	205.2	2.07	−22	NA
13	0	0	0	89	84	86	57.1	32	34.6	2.15	−29	−44
Median	0.7	2	1.45	92	75	78	138.0	78	52.5	2.53	−22	−34
Min	0.0	0.0	0.0	83.0	62.0	60.0	96.0	32.0	41.0	0.46	−65.0	−67.0
Max	2.9	2.9	10.7	144.0	87.0	91.0	141.0	247.0	64.0	5.24	11.0	−3.0

*BL* baseline, *ESA* erythropoiesis-stimulating agent, *max* maximum, *min* minimum, *NA* no assessment available, *PRBC* packed red blood cell, *pt* patient

^a^Change in PRBC transfusions 12 weeks before the first administration of ESA

^b^Change in PRBC transfusions 12 weeks after the first administration of ESA

^c^Change in the worst hemoglobin value and the highest reticulocyte count measured 6 weeks prior to the first administration of ESA

^d^Change in the worst hemoglobin value and the highest reticulocyte count measured 12 weeks after the first administration of ESA

^e^Percentage change in spleen volume from baseline to the last assessment prior to the first administration of ESA

^f^Percentage change in spleen volume from baseline to the first assessment after the first administration of ESA

In the 13 patients who received concomitant ruxolitinib and ESA, the worst hemoglobin value within 12 weeks of ESA administration improved in 3 patients, worsened in 2 patients, and did not change in 7 patients, compared with their worst pre-ESA study assessments; data were not available for 1 patient (Table 1). In patients with assessments at 12 weeks after the first ESA administration (n = 8), 6 patients had increases (median increase 7 g/L; range 5–57 g/L) and 2 patients had decreases in hemoglobin levels; 5 patients had no data available. The rate of PRBC transfusions per month within the 12 weeks before and after first ESA use (mean transfusion rates, 0.8 and 1.3, respectively) did not change for 1 patient, decreased for 2 patients, and increased for 3 patients (Table 1). Seven patients were transfusion independent at baseline and remained transfusion independent throughout this analysis (Table 1). Most patients (85 %) did not have any substantial change in reticulocyte counts within 12 weeks after ESA administration (Table 1).

The AEs reported in the +ESA group (n = 13) were similar to those previously reported in the overall ruxolitinib arm (n = 146) [12]. Six weeks prior to the first ESA administration, 10 of the 13 patients (77 %) had grade 3/4 hemoglobin abnormalities; 6 weeks after ESA use, most patients’ hemoglobin abnormalities improved to grade 2 [7/13 (54 %)], suggesting the concomitant use of an ESA and ruxolitinib was beneficial in some patients. Overall, serious AEs were reported for 8 of the 13 patients in the +ESA group (Table 2). Three patients experienced serious AEs (SAEs) prior to the first ESA administration, 2 experienced SAEs before and after the first administration, and 7 experienced SAEs after the first administration. One patient experienced a pulmonary embolism, potentially due to ESA use. Three events in 2 patients were possibly related to ruxolitinib: 1 patient experienced general health deterioration (grade 3) and respiratory tract infection (grade 4), and 1 patient experienced anemia (grade 3). There were 2 thromboembolic events; 1 patient had a pulmonary embolism (mentioned above), and a second patient had a grade 2 splenic infarct that occurred prior to ESA administration and was not suspected to be related to treatment.

**Table 2:** Serious adverse events regardless of relationship to study drug

	Preferred term	
Pt	Before ESA	After ESA
1	Cholesteatoma	
2	Respiratory tract infection	General physical deterioration^a^
		Respiratory tract infection^a^
		Renal failure, acute
5	NA	Hip fracture
		Septic shock
		Pneumonitis
		Depressed consciousness
6	Pulmonary arterial hypertension	Ascites
	Anemia^b^	Encephalopathy
	Varices esophageal	Enterococcal sepsis
	Upper gastrointestinal hemorrhage	
8	NA	Herpes zoster
		Pulmonary embolism
		Cardiac failure
9	NA	Abdominal pain
		Urinary tract infection
		Anemia^a,b^
		Radius fracture
		Lung infiltration
		Pneumonia
		Sepsis
		Renal failure
		Supraventricular tachycardia
		Hepatic failure
		Tachypnea
		Hyperuricemia
		Lumbar vertebral fracture
		Thoracic vertebral fracture
		Respiratory failure
10	NA	Traumatic fracture
13	NA	Gastroenteritis

*ESA* erythropoiesis-stimulating agent, *NA* not applicable (no SAE reported), *pt* patient, *SAE* serious adverse event

^a^Suspected by the investigator to have a relationship to the study drug

^b^Common Toxicity Criteria Grade 3 anemia

The use of an ESA did not appear to affect the efficacy of ruxolitinib with regard to splenomegaly. Twelve of the 13 patients (92 %) had spleen volume reductions from baseline with ruxolitinib treatment prior to ESA administration (Table 1). All 8 of 12 evaluable patients had sustained reductions after ESA administration, with 7 of these 8 patients showing a greater reduction in spleen volume after ESA use. Additionally, 1 patient experienced a reduction in spleen volume from baseline after ESA administration indicating a response to ruxolitinib (Table 1). These effects on spleen volume were not associated with an increased ruxolitinib dose; in fact, for most patients, the total daily dose of ruxolitinib remained the same or was lower after ESA administration.

As might be expected from what is known about JAK/STAT signaling [20], ruxolitinib-treated patients had higher median levels of erythropoietin than patients treated with BAT. The median levels of erythropoietin at baseline were similar in both the ruxolitinib (8.6 pg/mL, 95 % CI 7–13 pg/mL) and BAT (11.0 pg/mL, 95 % CI 7–21 pg/mL) arms. There was an initial rapid increase in erythropoietin levels in ruxolitinib-treated patients (150.0 % change from baseline at week 4), but no change was observed with BAT. Ruxolitinib-treated patients had higher median levels of erythropoietin than BAT-treated patients at week 24 (ruxolitinib, 36.0 pg/mL, 95 % CI 19–77 pg/mL; BAT, 8.7 pg/mL, 95 % CI 7–29 pg/mL) and even higher levels at week 48 [54.0 pg/mL, 95 % CI 31–98 pg/mL (216.3 % change) vs 26.0 pg/mL, 95 % CI 16–72 pg/mL (77.0 % change), respectively]. One explanation for the difference in erythropoietin levels between patients treated with ruxolitinib and BAT lies in the mode of action of both erythropoietin and ruxolitinib. Erythropoietin is the main regulator of erythropoiesis. Direct binding of erythropoietin to its receptor activates JAK2, which initiates signal transduction pathways that lead to the proliferation and terminal differentiation of erythroid precursor cells [20]. In patients receiving ruxolitinib, inhibition of JAK2 signaling may lead to impaired erythropoiesis and, potentially, anemia. The resulting decrease in the body’s oxygen levels causes circulating erythropoietin levels to increase up to 1000-fold in an attempt to boost the blood’s oxygen-carrying capacity by increasing red blood cell (and thus hemoglobin) concentration. Since ESAs share the same mechanism of action as endogenous erythropoietin, one might not expect treatment with an ESA to benefit patients with MF. Interestingly, it is the serum half-life of erythropoietin rather than the concentration that is mainly responsible for increased RBC concentrations, and recombinant ESAs currently in use have a prolonged terminal half-life compared with endogenous erythropoietin [20]. The prolonged half-life of ESAs in comparison to the relatively short half-life of ruxolitinib may be what aided some COMFORT-II patients in obtaining the clinical benefits observed in the treatment of anemia [20].

Despite these observed clinical benefits, previous studies have suggested that ESAs have limited activity in patients with MF with transfusion dependency, marked splenomegaly, serum erythropoietin level >125 U/L, or *JAK2* V617F homozygosity [16, 18]. One study evaluating the use of darbepoetin alfa in patients with MF, myeloid metaplasia, and anemia found that no patient with adequate erythropoietin levels (≈2–5 pM) responded to treatment [17]. Another study found that patients with primary MF (N = 43, Mayo Clinic, Rochester, MN) had a low overall response rate to treatment with an ESA (23 %) and that the response rate was not correlated with baseline serum erythropoietin level in any of the 43 patients (including transfusion-independent patients, n = 27), nor with primary MF-specific treatment history, use of concurrent cytoreductive therapy, cytogenetic findings, or *JAK2* V617F presence [16]. In addition to these findings, safety concerns such as the association of leukemic transformation with ESA use [16] have led investigators to recommend against ESA administration in patients with MF who are transfusion dependent or have a baseline hemoglobin ≥100 g/L [16, 17]. It can be noted that whereas the pathogenesis of MF-related anemia is complex, involving factors such as aberrant erythropoiesis and persistent low-grade hemolysis, ruxolitinib-associated anemia is self-limiting and dose dependent [21]; potential differences in the ability of an ESA to counteract MF-related and JAK inhibitor-mediated anemia may lie in this distinction [8, 11, 12, 22, 23]. Although the efficacy of an ESA to control JAK inhibitor-induced anemia in patients with MF is yet unknown, this analysis suggests that ESA use in this patient population is safe and could become a tool in helping patients achieve control of JAK inhibitor-induced anemia.

## Conclusions

In this report, the data suggest that concomitant use of an ESA with ruxolitinib is safe and does not affect the efficacy of ruxolitinib. However, the study is limited by the small number of patients analyzed and no definitive recommendations can be made at this time regarding the use of ESAs in the management of anemia in ruxolitinib-treated patients. Further investigations of ESAs in combination with ruxolitinib in this patient population are needed to help guide clinical decisions.

## Methods

COMFORT-II (ClinicalTrials.gov identifier, NCT00934544) is a randomized, open-label, multicenter phase 3 study comparing the safety and efficacy of ruxolitinib with BAT for the treatment of MF [12]. Patients with primary MF, post-polycythemia vera MF, or post-essential thrombocythemia MF classified as intermediate-2 or high risk by International Prognostic Scoring System criteria [1] were randomized 2:1 to receive ruxolitinib (15 or 20 mg twice daily, based on baseline platelet count [100–200 or >200 × 10^9^/L, respectively]) or BAT [1, 12]. Further study design details have been presented previously [12]. To prevent confounding efficacy analyses of spleen response, ESA use was discouraged but not prohibited [12]. ESAs were administered per investigator discretion based on joint recommendations from the American Society of Hematology and American Society of Clinical Oncology [15]. Transfusion rates were calculated as the PRBC units transfused per month within the 12 weeks before and after the first ESA administration. Spleen volumes were assessed by magnetic resonance imaging or computed tomography scan. Assessments were completed every 12 weeks in the core study until the primary endpoint was reached; responders were followed at 12-week intervals thereafter (data cutoff, 1 March 2012).

### Ethics, consent, and permissions

The study was sponsored by Novartis Pharmaceuticals and designed by Incyte. It was approved by the institutional review board at each participating institution and was conducted in accordance with the principles of the Declaration of Helsinki. All patients provided written informed consent.

## Abbreviations

AE: adverse event
BAT: best available therapy
COMFORT: Controlled Myelofibrosis Study with Oral JAK Inhibitor Treatment
ESA: erythropoiesis-stimulating agents
JAK: Janus kinase
MF: myelofibrosis
PRBC: packed red blood cell

## References

[1] Cervantes F, Dupriez B, Pereira A, Passamonti F, Reilly JT, Morra E (2009). New prognostic scoring system for primary myelofibrosis based on a study of the International Working Group for Myelofibrosis Research and Treatment. Blood.

[2] Cervantes F, Martinez-Trillos A (2013). Myelofibrosis: an update on current pharmacotherapy and future directions. Expert Opin Pharmacother.

[3] Levine RL, Gilliland DG (2008). Myeloproliferative disorders. Blood.

[4] Mesa RA (2010). Assessing new therapies and their overall impact in myelofibrosis. Hematology Am Soc Hematol Educ Prog.

[5] Jatiani SS, Baker SJ, Silverman LR, Reddy EP (2010). JAK/STAT pathways in cytokine signaling and myeloproliferative disorders: approaches for targeted therapies. Genes Cancer.

[6] Tefferi A (2013). Primary myelofibrosis: 2013 update on diagnosis, risk-stratification, and management. Am J Hematol.

[7] Mughal TI, Vaddi K, Sarlis NJ, Verstovsek S (2014). Myelofibrosis-associated complications: pathogenesis, clinical manifestations, and effects on outcomes. Int J Gen Med.

[8] Guglielmelli P, Vannucchi AM (2013). Struggling with myelofibrosis-associated anemia. Leuk Res.

[9] Tefferi A, Lasho TL, Jimma T, Finke CM, Gangat N, Vaidya R (2012). One thousand patients with primary myelofibrosis: the Mayo Clinic experience. Mayo Clin Proc.

[10] Harrison C, Mesa R, Ross D, Mead A, Keohane C, Gotlib J (2013). Practical management of patients with myelofibrosis receiving ruxolitinib. Expert Rev Hematol.

[11] Verstovsek S, Mesa RA, Gotlib J, Levy RS, Gupta V, DiPersio J (2012). A double-blind, placebo-controlled trial of ruxolitinib for myelofibrosis. N Engl J Med.

[12] Harrison C, Kiladjian JJ, Al-Ali HK, Gisslinger H, Waltzman R, Stalbovskaya V (2012). JAK inhibition with ruxolitinib versus best available therapy for myelofibrosis. N Engl J Med.

[13] Verstovsek S, Mesa RA, Gotlib J, Levy RS, Gupta V, DiPersio JF (2015). Efficacy, safety, and survival with ruxolitinib in patients with myelofibrosis: results of a median 3-year follow-up of COMFORT-I. Haematologica.

[14] Cervantes F, Vannucchi AM, Kiladjian JJ, Al-Ali HK, Sirulnik A, Stalbovskaya V (2013). Three-year efficacy, safety, and survival findings from COMFORT-II, a phase 3 study comparing ruxolitinib with best available therapy for myelofibrosis. Blood.

[15] Rizzo JD, Brouwers M, Hurley P, Seidenfeld J, Arcasoy MO, Spivak JL (2010). American Society of Hematology/American Society of Clinical Oncology clinical practice guideline update on the use of epoetin and darbepoetin in adult patients with cancer. Blood.

[16] Huang J, Tefferi A (2009). Erythropoiesis stimulating agents have limited therapeutic activity in transfusion-dependent patients with primary myelofibrosis regardless of serum erythropoietin level. Eur J Haematol.

[17] Cervantes F, Alvarez-Larran A, Hernandez-Boluda JC, Sureda A, Granell M, Vallansot R (2006). Darbepoetin-alpha for the anaemia of myelofibrosis with myeloid metaplasia. Br J Haematol.

[18] Tsiara SN, Chaidos A, Bourantas LK, Kapsali HD, Bourantas KL (2007). Recombinant human erythropoietin for the treatment of anaemia in patients with chronic idiopathic myelofibrosis. Acta Haematol.

[19] Tefferi A (2011). How I treat myelofibrosis. Blood.

[20] Elliott S, Pham E, Macdougall IC (2008). Erythropoietins: a common mechanism of action. Exp Hematol.

[21] Mesa RA, Cortes J (2013). Optimizing management of ruxolitinib in patients with myelofibrosis: the need for individualized dosing. J Hematol Oncol.

[22] Quintas-Cardama A, Kantarjian H, Cortes J, Verstovsek S (2011). Janus kinase inhibitors for the treatment of myeloproliferative neoplasias and beyond. Nat Rev Drug Discov.

[23] Gotlib J (2013). JAK inhibition in the myeloproliferative neoplasms: lessons learned from the bench and bedside. Hematology Am Soc Hematol Educ Prog.

